# Dental findings on face and neck imaging

**DOI:** 10.1590/0100-3984.2019.0104

**Published:** 2021

**Authors:** Isabela dos Santos Alves, Daniela Ferreira Vieira Vendramini, Claudia da Costa Leite, Eloisa Maria Mello Santiago Gebrim, Ula Lindoso Passos

**Affiliations:** 1 Hospital Sírio-Libanês, São Paulo, SP, Brazil.; 2 Departamento de Radiologia, Faculdade de Medicina da Universidade de São Paulo (FMUSP), São Paulo, SP, Brazil.

**Keywords:** Dental arch/pathology, Periodontal diseases/diagnosis, Tomography, X-ray computed, Magnetic resonance imaging, Arcada dentária/patologia, Doenças periodontais/diagnóstico, Tomografia computadorizada, Ressonância magnética

## Abstract

When it is necessary to evaluate dental structures, the typical method is to obtain intraoral or panoramic X-rays at specialized dental clinics. However, in the daily practice of head and neck radiology, or even general radiology, it is common to encounter clinical situations or examination findings related to dental problems that should not be ignored. Because such problems can often be responsible for the clinical complaints of patients, this review aims to assist radiologists in identifying and describing common dental conditions on computed tomography of paranasal sinuses, face, and neck. It is important for radiologists to have knowledge of dental arch anatomy and its relationships with facial structures, as well as of major dental pathologies, including periapical sclerotic lesions, odontogenic cysts, fistulas, and abscesses, together with knowledge of incidental findings without clinical repercussions, which should be easily identified and stressed by the radiologist when necessary. The imaging methods most commonly used in evaluation of paranasal sinuses and face are computed tomography and magnetic resonance imaging. Those methods allow radiologists to recognize and become familiar with the main dental findings. The description of such findings by a radiologist can lead to a change in treatment strategy.

## Abstract (pt)

A avaliação das estruturas dentárias, quando necessária, é realizada na maioria das vezes com radiografias intraorais e panorâmicas em clínicas radiológicas especializadas em odontologia. No entanto, na prática do radiologista de cabeça e pescoço e mesmo do radiologista geral, é comum identificarem-se alterações odontológicas que não devem passar despercebidas. Este artigo de revisão tem como objetivo auxiliar o radiologista a identificar e descrever afecções dentárias comuns e, muitas vezes, responsáveis por frequentes queixas clínicas, nas tomografias computadorizadas de face e pescoço. O conhecimento da anatomia da arcada dentária, as relações com as estruturas da face, bem como o domínio das principais doenças odontológicas, como cáries, lesões escleróticas periapicais, cistos odontogênicos, fístulas, abscessos e outros achados incidentais sem repercussão clínica, devem ser prontamente identificados e valorizados pelo radiologista. Os métodos de imagem mais comumente utilizados na avaliação dos seios paranasais e face são a tomografia computadorizada e a ressonância magnética. Esses exames possibilitam a identificação e familiaridade com os principais achados dentários, que devem ser descritos para auxiliar na abordagem do paciente.

## INTRODUCTION

Imaging examinations play an important role in the diagnosis and follow-up of disorders of the face and neck. Even when requested for purposes other than that of obtaining a dental image, computed tomography (CT) and magnetic resonance imaging (MRI) can contribute to the incidental identification of such disorders.

Teeth are vital for the basic functions of eating and speaking. When interpreting CT and MRI scans of the head and neck, the radiologist can identify dental lesions, thus preventing the progression of a disease and its complications^(1)^.

This article aims to review the dental anatomy and its relationships with the cervical spaces, in order to help the general radiologist identify the main dental pathologies that can be found in CT and MRI scans, discuss the imaging findings that are essential for the diagnosis (and differential diagnosis) of dental disorders, and identify any associated complications.

## ANATOMY OF THE ORAL AND DENTAL CAVITIES

**Figure 1:**
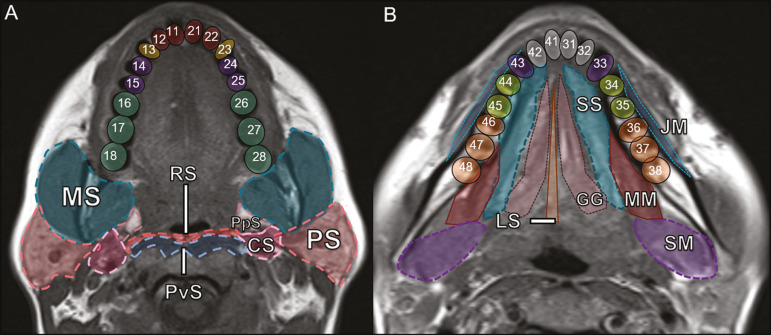
Tooth count and anatomical relationships between teeth. A: With adjacent cervical spaces. B: With the floor of the mouth. CS, carotid space; MS, masticatory space; PS, parotid space; PpS, parapharyngeal space; PvS, prevertebral space; RS, retropharyngeal space; GG, genioglossos muscle; JM, jugal mucosa; MM, mylohyoid muscle; SS, sublingual space; SM, submandibular space; LS, lingual septum.

It is fundamental for radiologists to be familiar with the anatomy of the oral cavity to allow a detailed description of dental lesions and their relationship with adjacent structures. The oral cavity is the ventral part of the aerodigestive tract. Its main structures are the lips, anterior two thirds of the tongue, oral mucosa, floor of the mouth, hard palate, mandibular/maxillary alveoli (including the teeth), retromolar trigone (mucosa posterior to the third molar that covers the ascending branch of the mandible), sublingual space, and submandibular space^(2)^. The anterior limit is the lips, and the posterior limit is the virtual line that passes through the circumvallate papillae, the anterior tonsillar pillars, and the junction between the hard and soft palate. The lateral walls are formed by the cheeks (jugal or vestibular mucosa) and the retromolar trigones. The superior limit is bounded by the hard palate and its lower portion is composed of the floor of the mouth, which is bounded by the mylohyoid muscle^(3)^. Knowledge of the relationships that the teeth have with the structures of the oral cavity and cervical spaces is essential for defining the extent of a lesion (Figure 1).

The teeth are situated in the alveolar processes of the maxilla and mandible. Each tooth has an internal lingual surface and an external facial (buccal) surface. An adult has 32 permanent teeth that are divided into four quadrants (Figure 1). In each quadrant, from the central teeth to the external teeth, in the maxilla as well as in the mandible, there is one central incisor, a lateral incisor, a canine, two premolars, and three molars^(4)^.

**Figure 2:**
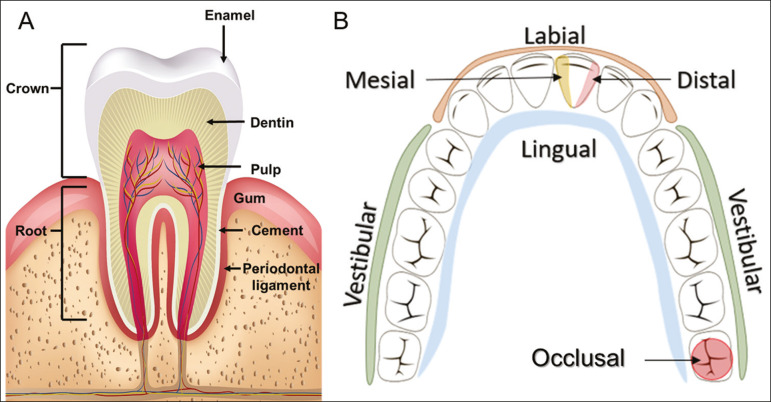
Tooth anatomy. A: Schematic drawing. B: Dental surfaces.

The dental crown is visible above the gum line, and the alveolar component is known as the root or radicle (Figure 2A). The densely mineralized surface of the crown is called the enamel, under which is the dentin, which is the largest component of the tooth. The pulp is the central chamber of the tooth, containing blood vessels and nerves, and shows low attenuation on CT^(5,6)^. The cement is the intra-alveolar extension of the enamel (it protects the root) and is connected to the maxilla by a fibrous periodontal ligament, constituting a space for the potential dissemination of disease. To better characterize the pathological processes that affect the tooth, it is important to know the tooth surfaces, which are determined by the structures in contact with them: labial (in contact with the labial mucosa); vestibular (in contact with the buccal mucosa); lingual (in contact with the tongue); and occlusal (the masticatory or chewing surface of premolars and molars facing the antagonist arch). In addition, each tooth has a mesial surface, which faces toward the midline, and a distal surface, which faces away from the midline^(4)^, as shown in Figure 2.

## DENTAL ALTERATIONS ON IMAGING EXAMINATIONS

### Supernumerary teeth

**Figure 3:**
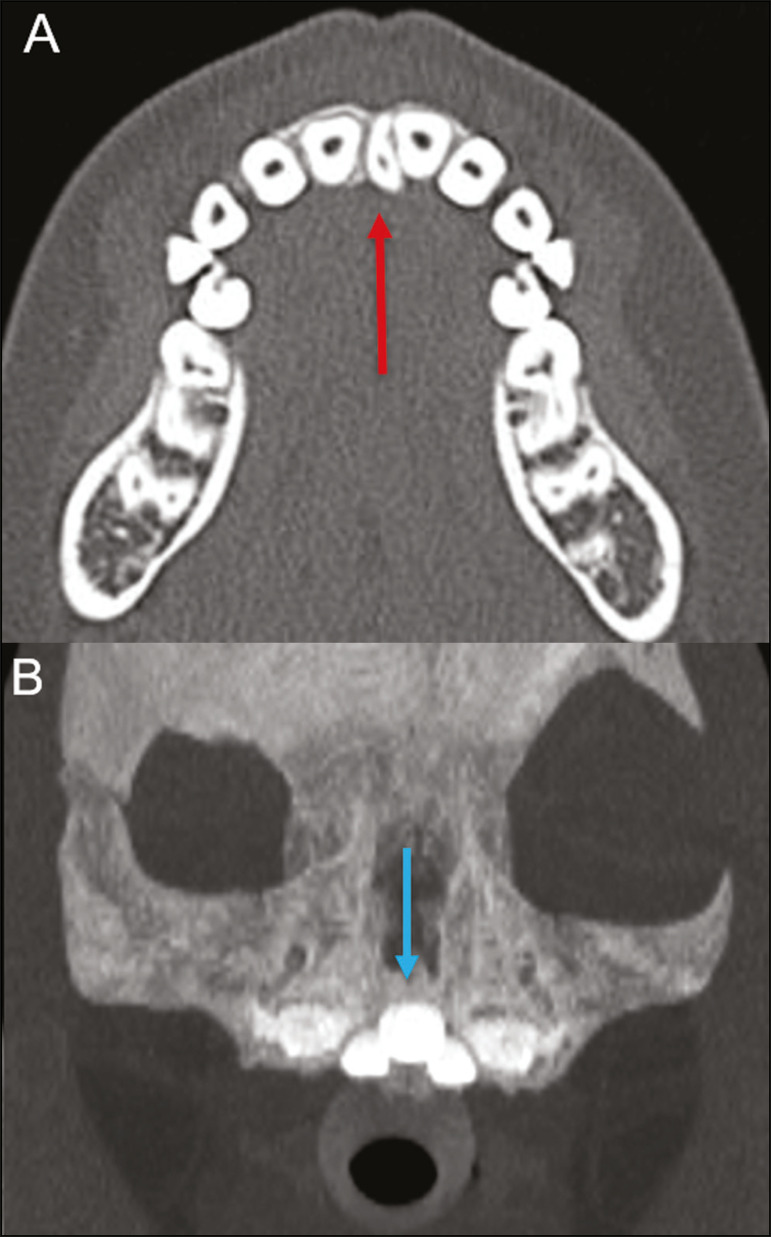
Axial (A) and coronal in maximum intensity projection (B) CT scans showing dental supernumerary element impacted in the midline of the maxila between two central incisors-a *mesiodens* (red arrow)-which differs from the single central incisor (blue arrow).

The presence of extra teeth, a condition also known as hyperdontia, is defined as the existence of an excessive number of teeth in relation to the normal permanent dentition. These extra teeth can develop in the upper or lower dental arch. If they are similar to the normal teeth, they are called supernumerary teeth: a tooth located in the midline between the two central incisors is known as a *mesiodens*; a tooth that erupts ectopically in the premolar region is known as a parapremolar; and a supernumerary molar posterior to the third molar is known as a distomolar. A *mesiodens* is a supernumerary tooth and should be distinguished from the solitary median maxillary central incisor syndrome, characterized by the presence of a single upper central incisor that erupted precisely in the midline and is associated with defects in the development of the midline (Figure 3). Supernumerary teeth can cause alterations in adjacent teeth, dental retention, or delayed/ectopic eruption^(7)^.

### Impacted teeth

**Figure 4:**
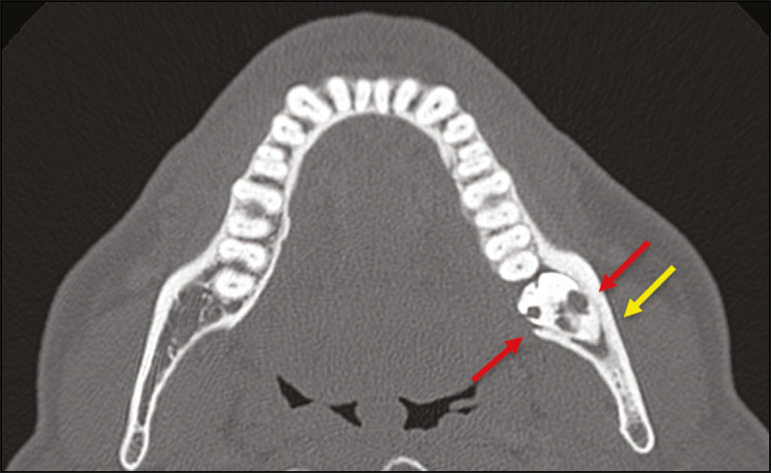
Axial CT of the paranasal sinuses showing lower left third molar (tooth 38) partially unerupted, impacted at the distal root of the second molar, showing cariogenic lesions (red arrows) together with signs of periodontitis (yellow arrow).

**Figure 5:**
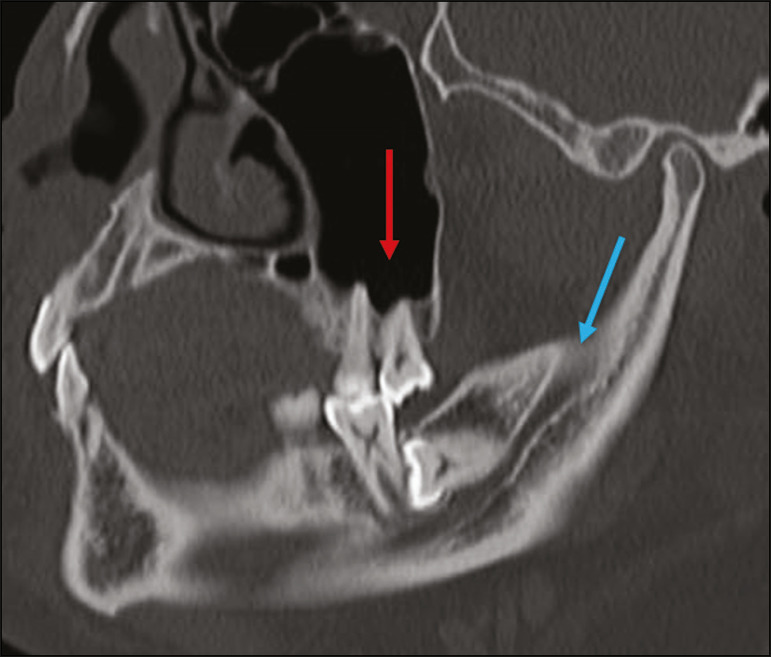
Sagittal CT of the paranasal sinuses showing the relationship between the maxillary dental arch and the floor of the maxillary sinus (red arrow), as well as the relationship between an impacted third molar (in the mandibular dental arch) and the mandibular canal where the inferior alveolar nerve passes (blue arrow).

Impaction is a pathology in which the tooth does not reach its functional position. The tooth can be completely or partially unerupted (intraosseous or subgingival) or positioned against another tooth, bone, or soft tissue, so that its eruption is unlikely. In clinical practice, the most common condition is an impacted third molar (Figure 4), which can remain asymptomatic or promote the appearance of other conditions, such as cavities, pericoronitis, cysts, tumors, and radicular resorption of the tooth^(8,9)^. The report should detail the relationship of the impacted tooth with the adjacent teeth-the mandibular canal inferiorly or the sinus floor superiorly (Figure 5).

### Dental caries

Dental caries are caused by acids produced by bacteria due to fermentation of carbohydrates, which results in a drop in pH on the tooth surface. As a response to this aggression, calcium and phosphate ions leach out of the enamel, resulting in demineralization and erosion of the dental surface reaching the dentin-enamel junction and giving the characteristic aspect seen on imaging scans^(10,11)^.

On CT, dental caries appear as rounded, focal, hypodense areas in the dentin, extending from the tooth surface, generally in a mushroom shape, the "stalk" represented by a narrow channel through the enamel and the "cap" represented by a larger region of demineralized dentin (Figure 4). Dental caries can be single or multiple and, when severe, can be accompanied by periapical and periodontal disease, culminating in tooth loss. On CT, occlusive caries (those on the masticatory surface), are best visualized in the sagittal and coronal planes, whereas proximal or non-occlusive (vestibular, lingual, mesial, or distal) caries are more easily identified in the axial and coronal planes^(1,11)^.

### Periodontal disease and periapical pathologies

Periodontal diseases occur as a function of inflammatory and immunological reactions in the periodontal tissues induced by microorganisms in the dental biofilm (bacterial plaque). Plaque damages the connective tissue and the alveolar bone, destroying the periodontal ligaments. On CT, an increase of the periodontal ligament space can be seen, as can bone loss between teeth and at the furcations between roots^(1)^.

Apical periodontitis is a chronic inflammatory disorder of the periradicular tissues caused by etiologic agents of endodontic origin, and it can also develop as a consequence of a secondary infection subsequent to an endodontic procedure^(1)^. It occurs around the dental apex, where dynamic factors between bacterial agents and the defenses of the host in the interface between the infected radicular pulp and the periodontal ligament cause local inflammation, resorption of hard tissues, destruction of periapical tissue, and formation of different histopathological categories of apical periodontitis such as periapical granuloma, periapical abscess, and periapical cyst-also known as radicular cyst^(11-13)^. A granuloma is the product of the formation of reactive granulation tissue, without an accompanying infectious process, together with a thin fibrous capsule surrounding the periapical lesion^(14)^.

**Figure 6:**
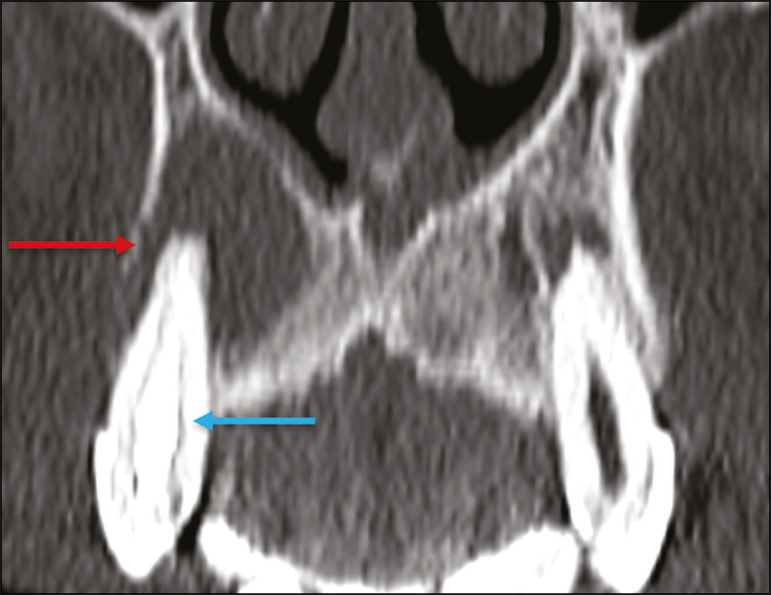
Coronal CT of the paranasal sinuses showing periapical disease with the formation of a periapical cyst at the upper right canine (red arrow), which presents signs of endodontic treatment (blue arrow).

A periapical cyst is a cavity lined with epithelium that contains fluid or semisolid material and is commonly surrounded by dense connective tissue and inflammatory infiltration. It can be difficult to distinguish between a periapical cyst and a granuloma on imaging; the granuloma is small with ill-defined margins, whereas the cyst is typically larger with well-defined borders^(1,11,12)^. On CT, most such cysts appear as unilocular lesions in the periapical region, are smaller than 1 cm, are rounded or pear-shaped (Figure 6), and are delimited by a thin border of cortical bone. On MRI, they present a hypointense signal on T1-weighted images and a hyperintense signal on T2-weighted images, with no enhancement on paramagnetic contrast-enhanced images^(1,11)^.

The term periapical abscess can be used when a granuloma or cyst is infected with bacteria originating from the necrotic pulp. An abscess is characterized by a rapid onset of spontaneous pain, tooth sensitivity to pressure, the formation of pus, and swelling of the adjacent soft tissues. In the initial stages, there is pain to the extent that the pressure accumulates in the bony alveolus. Perforation of the cortical plate can result in accumulation of pus under the periosteum. Perforation of the periosteum leads to drainage of the infected material into the adjacent soft tissues or gingival mucosa, which results in a reduction in pain^(11)^.

### Diseases secondary to dental disorders and their complications

#### Oroantral fistula

An oroantral fistula is a connection between the dental alveolus and the maxillary sinus and can form as a complication of dental extraction, periodontal disease, or endodontic disease. Due to the proximity of the roots of the teeth to the maxillary sinus floor, bone erosion can occasionally allow an infection to infiltrate the maxillary antrum.

On CT, discontinuity of the maxillary sinus floor can be identified and can be accompanied by mucosal thickening, with or without obliteration of the corresponding sinus. The dimensions of the bone defect should be included in the radiological report^(15,16)^. The routine evaluation of the radicular apices and of periodontal integrity on CT scans of the paranasal sinuses prevents these infectious foci from being overlooked^(10,11)^. To better characterize a fistula, the puffed-cheek maneuver can be used during the image acquisition; this entails asking patients to fill their mouth with air during the scanning in order to distend the oral cavity and separate the mouth mucosa from the gingival mucosa, thus increasing accuracy in the location of mucosal lesions and small abscesses. In the evaluation of an oroantral fistula, using this maneuver can reveal the passage of air from the oral cavity into the maxillary sinus through the fistula^(17)^.

#### Odontogenic sinusitis

**Figure 7:**
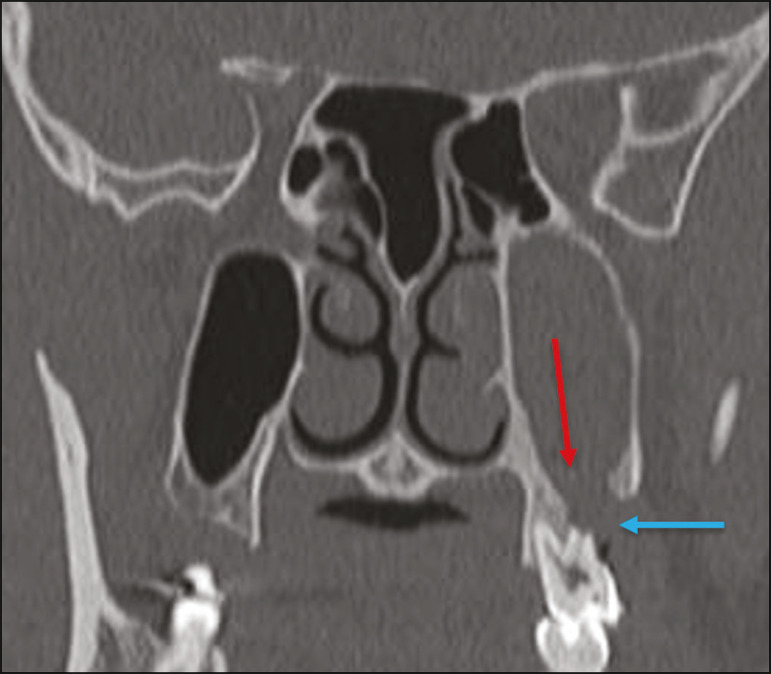
Coronal CT of the paranasal sinuses showing a bone defect in the floor of the left maxillary sinus (red arrow), which corresponds to an oroantral fistula causing odontogenic sinusitis. Note also the radicular amputation of the adjacent tooth (blue arrow).

Odontogenic disease is thought to be responsible for 10-12% of cases of maxillary sinusitis, especially when unilateral (Figure 7). Periodontal disease doubles the risk of developing maxillary sinusitis, due to the anatomical proximity of the radicular apices to the maxillary sinuses. Radicular apices are typically separated from the sinuses by a distinct bony partition. In one third of the population, however, that partition can be absent in the region of the first and second molar, the maxillary antrum being separated only by mucosa^(18-20)^.

Distinguishing between the sinusitis caused by obstruction of the ostia and odontogenic sinusitis is important in determining the most appropriate treatment for patients. Failure to identify odontogenic disease results in recurrent sinusitis because the microorganisms related to dental conditions are different from those isolated in cases of non-odontogenic sinusitis. The combination of findings related to periodontal and apical diseases, together with the presence of a defect in the maxillary sinus floor, is highly suggestive of a causal relationship and should be clearly communicated in the radiological report^(18-20)^.

#### Osteomyelitis

A dental infection in a context of inadequate treatment can result in bone infection. Other potential sources of dissemination are fractures, particularly those not recognized or healed, which expose the medullary cavity to periodontal pathogens or pathogens from the oral cavity. Acute suppurative odontogenic osteomyelitis generally does not demonstrate characteristic imaging findings, whereas chronic osteomyelitis causes a great variety of bone reactions^(21,22)^. Osteomyelitis can become chronic if the initial infection is subclinical or goes untreated, evolving in some cases to cortical bone rarefaction, formation of fistulous tracts, extraosseous abscesses, or subperiosteal abscesses^(1,11)^.

**Figure 8:**
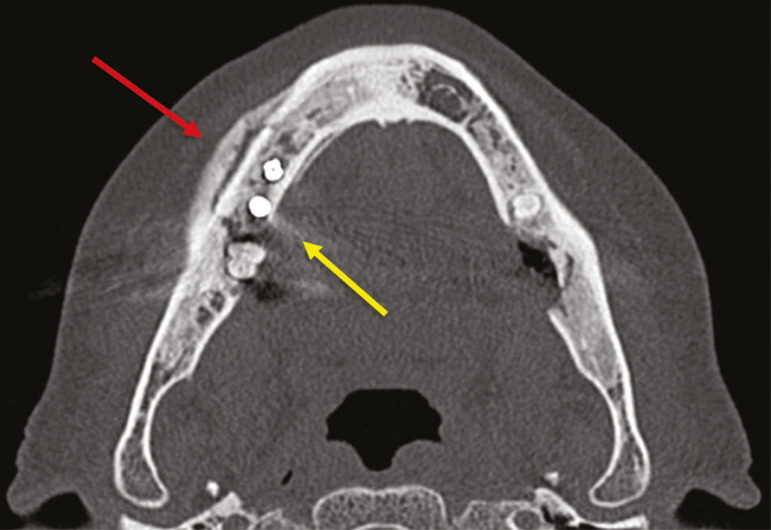
Axial CT of the face showing a periosteal reaction in the right branch of the mandible, together with bone sclerosis (red arrow). Note the areas of erosion areas throughout the lingual cortex on the right (yellow arrow). Taken together, these findings are suggestive of osteomyelitis.

Findings of chronic osteomyelitis on CT are lytic lesions (representing areas of osteonecrosis) and an accumulation of pus, surrounded by sclerosis of the bone marrow. Vestibular or lingual cortical dehiscence can also be seen, together with cortical thickening or a periosteal reaction (Figure 8). In addition, there can be extensive inflammatory thickening of the adjacent soft tissues, mimicking a neoplasm^(11,23)^.

Other frequent findings, in addition to peripheral sclerosis and a mixed pattern of trabecular osteolysis, are fragmentation with sequestration and expansion of surrounding bone. On MRI, a marked hypointense signal is seen on T1-weighted images, with intense enhancement on gadolinium contrast-enhanced images. Small abscesses can restrict the diffusion of water molecules^(1,24)^.

#### Ludwig's angina

Ludwig's angina is a potentially life-threatening necrotizing acute cellulitis that involves the floor of the mouth. Edema appears with rapid and progressive enlargement of cervical soft parts, resulting in compression of the airways. Although more than 90% of cases of Ludwig's angina are odontogenic, it can also result from penetrating trauma^(25,26)^.

Ludwig's angina is caused by aerobic and anaerobic bacteria, with the most common being *Streptococcus viridans, Staphylococcus aureus, Fusobacterium nucleatum, Peptostreptococcus* spp., *Enterobacter aerogenes*, and B-hemolytic streptococci of the genus *Bacteroides*^(21,26)^.

**Figure 9:**
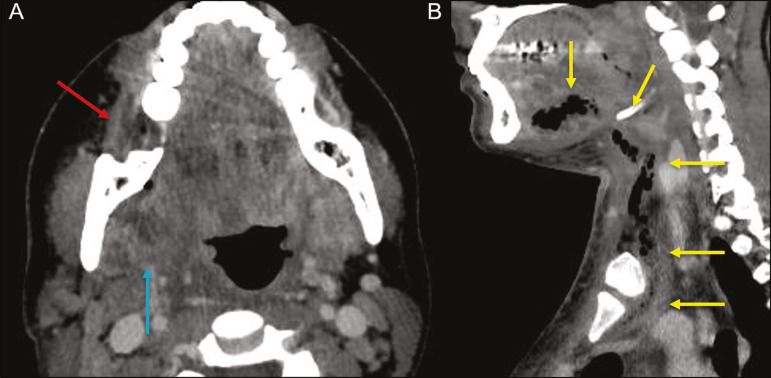
Axial and sagittal CT scans of the face and neck (A and B, respectively) of a patient submitted to extraction of lower right third molar who evolved to the formation of an odontogenic abscess (red arrow). The fluid collection extended to the masticatory space (blue arrow) and, subsequently, to the remaining deep cervical spaces (yellow arrows), to the level of the mediastinum, causing mediastinitis. These findings are compatible with Ludwig's angina.

This condition mainly originates in the lower second and third molars because their apices extend down to near the mandibular insertion of the mylohyoid muscle (mylohyoid line), which is responsible for acting as a barrier in the oral cavity and for preventing infections from spreading to the other cervical spaces^(1,11,21,26)^. Depending on the tooth affected, there is a predictable pattern of dissemination in the cervical spaces. When infected, the lower third molar drains into the submandibular space and the other lower teeth drain into the sublingual space. In some cases, the infection can extend to the mediastinum, resulting in mediastinitis^(1,11)^, as depicted in Figure 9.

**Figure 10:**
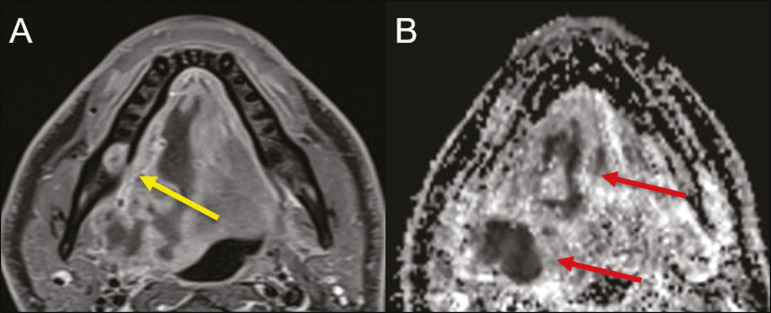
Contrast-enhanced axial T1-weighted MRI sequence of the face (A) and apparent diffusion coefficient map (B) showing extraction (A) of the lower right second molar (yellow arrow) with granulation tissue and signs of alveolar inflammation accompanied by a heterogeneous fluid collection showing restricted diffusion (B), delineating the planes of the floor of the mouth, and extending to the base of the tongue, causing narrowing of the oropharyngeal air column (red arrows).

On CT and MRI, an inflammatory reaction is identified by the densification of adjacent fat and a change in signal intensity, respectively, characterizing edema and soft tissue enlargement. In a more advanced phase, a fluid collection with peripheral enhancement can be identified, characterizing an organized abscess. Although Ludwig's angina is uncommon, it is a clinical emergency. The radiologist should evaluate the degree of airway involvement and the extent of the inflammatory process in adjacent structures, as well as locating the drainable abscess and the odontogenic focus involved in the process^(1,11,21,25)^, as shown in Figure 10.

### Dental trauma

Traumatic dental injuries occur with great frequency in children and young adults. Dislocations are the most common in primary dentition, whereas crown fractures are more common in permanent dentition. An appropriate diagnosis, as well as the appropriate planning and follow-up of treatment, is essential to guarantee a favorable outcome. Children who present to an emergency department with facial fractures are likely to present dental injuries^(27-29)^.

#### Tooth fracture

**Figure 11:**
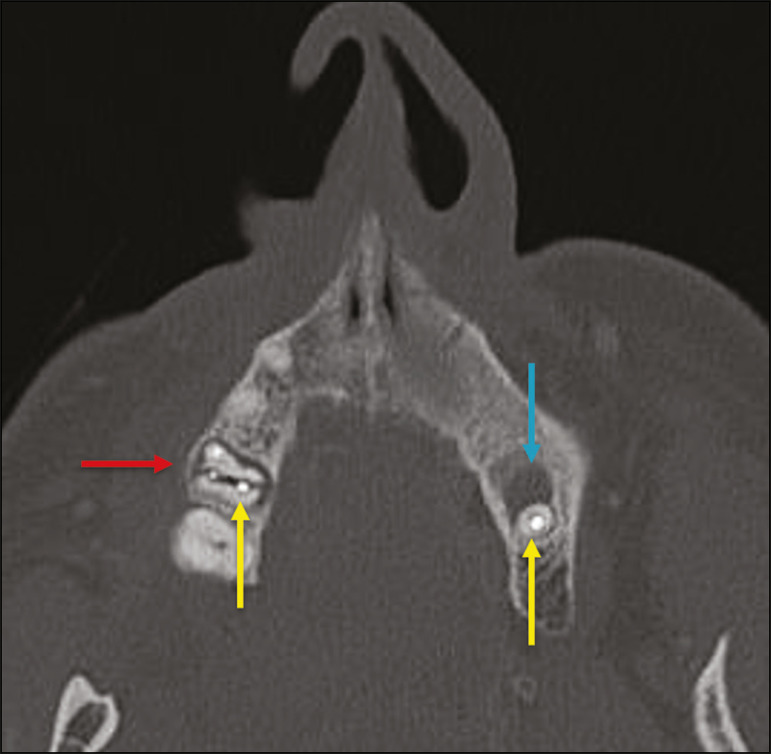
Axial CT of the face showing a longitudinal fracture of the upper right first molar (red arrow) with signs of endodontic treatment (yellow arrows) in some teeth. Note also the periapical disease in the upper left first molar (blue arrow).

Fractures affecting different segments of the tooth have specific causes and findings, as described below (Figure 11):

- Crown - This would require a great impact to the tooth, enough to break the enamel, the dentin, or both, and possibly the pulp as well^(27-29)^.
- Root - This happens more in patients undergoing endodontic treatment. Imaging characteristics are a radiolucent line between the fragments, an abnormal shape contour, and discontinuity of the root in the periodontal ligament space^(28)^.
- Alveolar process - This generally involves the lingual surface, vestibular surface, or both. Most of these fractures are accompanied by dental injuries that typically affect the anterior teeth back to the premolars and are usually accompanied by dislocation^(28)^.

#### Dental dislocation

**Figure 12:**
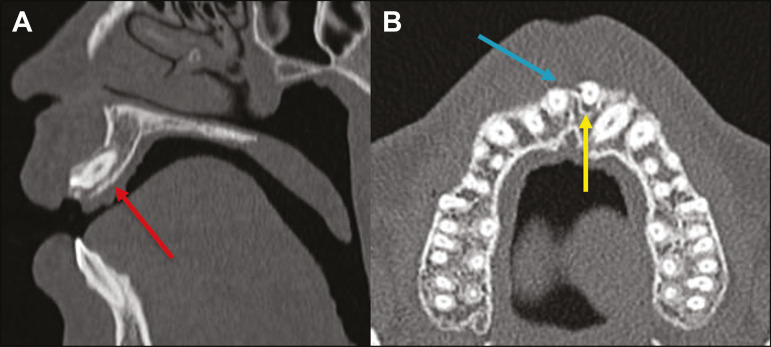
Sagittal and axial CT scans of the face (A and B, respectively) showing partial loss of the crown with root intrusion in the upper left lateral incisor (A) and a hypodense halo around the root of the upper left central incisor (B), with anterior dislocation of that tooth, suggestive of dislocation (yellow arrow), together with traces of fractures in the alveolar process in the maxila between the central incisor and the upper left lateral incisor (blue arrow).

Dental dislocation is a general term that encompasses various types of injuries such as concussion, subluxation, intrusive or extrusive luxation, and lateral luxation or avulsion, which is the complete dislocation of a tooth out of its alveolus (Figure 12). The most affected structure is the periodontal ligament space. The radiologist should identify and report the different types of dental dislocations^(27,28)^, as detailed in Table 1.

**Table 1:** Types of dental dislocations.

Type of dislocation	Description	Imaging finding
Concussion	Inflammation of the periodontal ligament: increasing dental sensitivity to percussion. There are no significant dislocations	No abnormalities; potential for the development of pulpal necrosis over time
Subluxation	Lesion in the dental support structures, without dislocation	Widening of the periodontal ligament space
Intrusive luxation (Figure 12A)	Dislocation of the tooth to within the alveolar process along the axis of the tooth; considered complete when the tooth is completely encapsulated by surrounding tissues and partial when the tip of the crown is still visible	Reduction or absence of the radicular periodontal ligament space
Extrusive luxation (Figure 12B)	Partial dental dislocation to outside the alveolar process	Overall increase in the radicular periodontal ligament space
Lateral luxation	Eccentric dislocation (in a non-craniocaudal direction) accompanied by a comminuted fracture of the alveolar process	Widening of the radicular periodontal ligament space
Avulsion	Complete dislocation of the tooth to outside the alveolar space	Tooth no longer within the alveolus

## DENTAL TREATMENTS

### Root canal

A root canal is an endodontic technique to treat dead or infected pulp in order to prevent and treat apical periodontitis. It eliminates microorganisms and the necrotic tissue by chemical and mechanical debridement with the appropriate radicular filling to prevent reinfection. Once the canals have been emptied and cleaned, they are filled in with dental cement or, more commonly, with a compound of zinc oxide and gutta-percha, a natural nontoxic latex with antimicrobial properties. On CT, this material can be seen as a hyperdense area within the dental pulp (Figure 11). The most common complications related to root canal treatment include extravasation of material, dentoalveolar abscess, pericementitis, and crown-root fracture^(1,30)^.

### Implants

Dental implants are used in order to replace absent teeth and provide more stability than a removable prosthesis. Candidates for dental implants are evaluated pre-surgery to determine if the alveolar process of the maxilla or mandible can receive an implant. The thickness of the alveolar bone is variable and edentulous regions suffer atrophy and bone resorption due to disuse. Occasionally, it is necessary to have bone grafts to increase the amount of available bone for the implants. Patients should also be evaluated to determine the precise location of the mandibular canal (neurovascular bundle), maxillary sinuses, and incisor foramen, so as to minimize the risk of complications^(31-33)^.

Dental implants are made of titanium and are fused to the mandible through the growth of osteoblasts-a process called osseointegration. The implant is inserted in the edentulous area to provide anchoring for a dental prosthesis. It has the shape of a cylinder or screw and functions as the root of a tooth. There are two main types of implants^(32,33)^: intraosseous implants, which are inserted surgically directly into the bone; and subperiosteal implants, which are made from a metal structure attached to the alveolar surface below the gum line. Another type of implant is a zygomatic implant, which is used as an alternative to a bone graft to rehabilitate the edentulous atrophic maxilla or in cases of extensive bone defects in the maxilla^(33)^.

## CONCLUSION

The main dental disorders identifiable on CT and MRI should be recognized and described by radiologists. The identification of dental and periodontal diseases on imaging scans has the potential to alter the management of these cases and prevent complications such as sinus diseases, chronic periodontitis, abscesses, nonunion fractures, osteomyelitis, and oroantral fistulas. Early diagnosis, immediate dental referral if necessary, and the appropriate treatment of these patients are important to prevent tooth loss and potentially life-threatening complications.
